# Supplementary material for: Discordant gene responses to radiation in humans and mice and the role of hematopoietically humanized mice in the search for radiation biomarkers
Source: Sci Rep. 2019 Dec 19;9:19434. doi: 10.1038/s41598-019-55982-2 (PMC6923394; doi:10.1038/s41598-019-55982-2)
Supplement: Supplementary file 3 — Supplementary Information3 [file 41598_2019_55982_MOESM3_ESM.pdf]

Discordant gene responses to radiation in humans and mice and the role of hematopoietically humanized mice in the search for radiation biomarkers.

Shanaz A. Ghandhi1*, Lubomir Smilenov1, Igor Shuryak1, Monica Pujol-Canadell1, Sally A Amundson1

The [networks, functional analyses, etc.] were generated through the use of IPA (QIAGEN Inc., https://www.qiagenbioinformatics.com/products/ingenuity-pathway-analysis).

Reference: Bioinformatics. 2014 Feb 15;30(4):523-30. doi: 10.1093/bioinformatics/btt703. Epub 2013 Dec 13.

Causal analysis approaches in Ingenuity Pathway Analysis.

Krämer A[1], Green J, Pollard J Jr, Tugendreich S.

p53 Homologene Hu

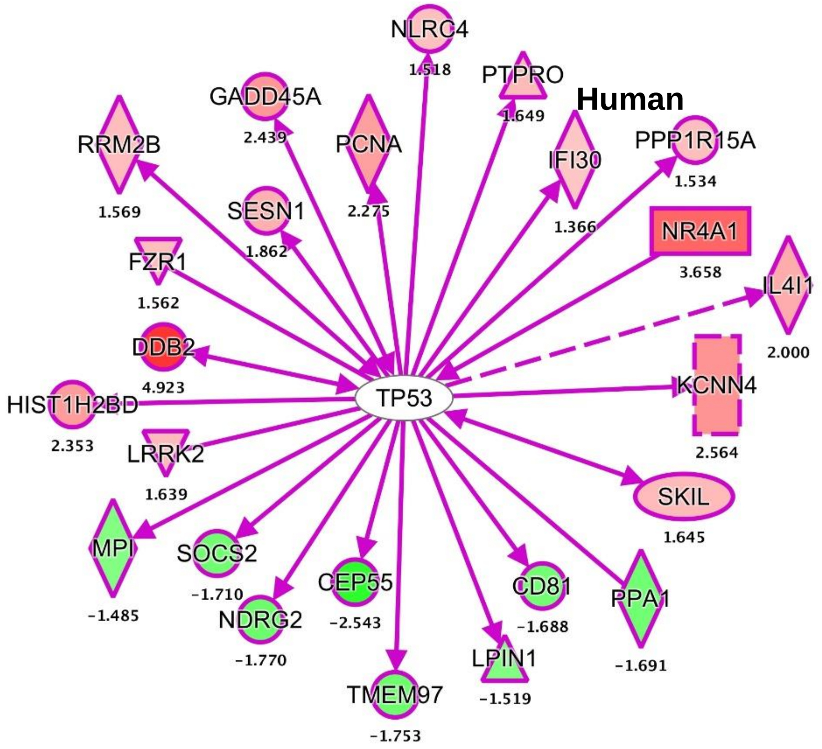

© 2000-2018 QIAGEN. All rights reserved.

p53 Homologene Hu

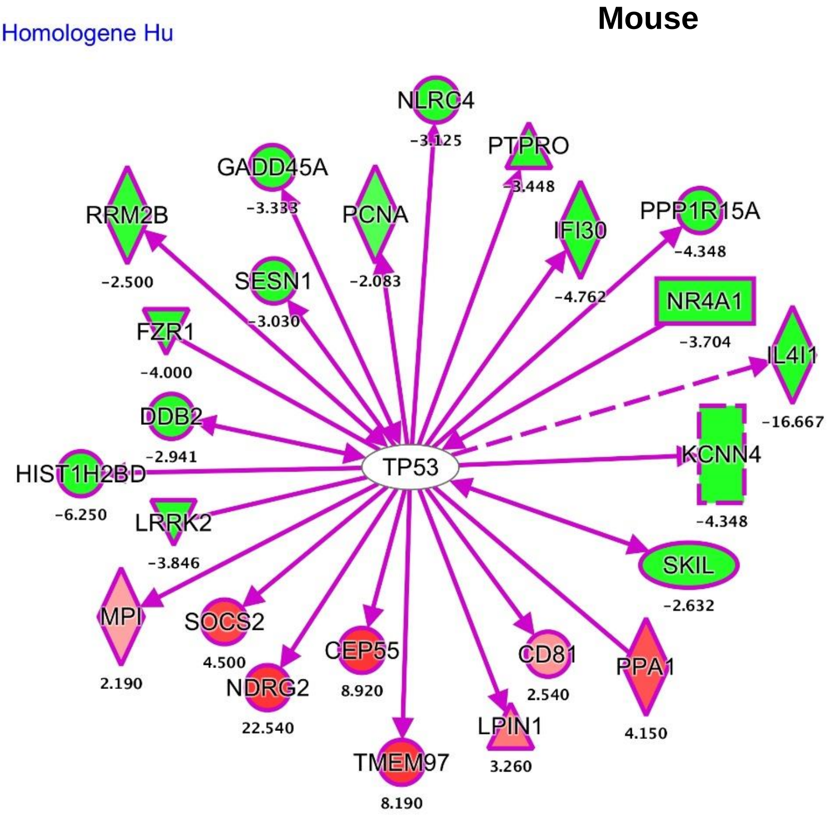

© 2000-2018 QIAGEN. All rights reserved.
